# RETRACTION: Exosomes Derived From Human Umbilical Cord Wharton's Jelly Mesenchymal Stem Cells Ameliorate Experimental Lymphedema

**DOI:** 10.1002/ctm2.70511

**Published:** 2025-10-27

**RETRACTION: **Ting Z, Zhi‐xin Y, You‐wen Y, et al. Exosomes derived from human umbilical cord Wharton's Jelly Mesenchymal stem cells ameliorate experimental lymphedema. *Clinical and Translational Medicine* 2021;11(7):e384. https://doi.org/10.1002/ctm2.384.

The above article, published online on 19 July 2021 in Wiley Online Library (wileyonlinelibrary.com), has been retracted by agreement between the journal Editor‐in‐Chief, Xiangdong Wang; the Shanghai Institute of Clinical Bioinformatics; and John Wiley & Sons Australia, Ltd.

The retraction has been agreed due to concerns raised by third parties. Specifically, instances of image duplication were detected within Figure 2H. Further investigation by the publisher detected additional instances of image duplication between Figures 1L and 3G, and between Figures 1J and 3H. The clarification and data provided by the authors was insufficient to address the concerns, and the extent of the detected issues undermined the editors’ trust in the accuracy and integrity of the whole body of work. Accordingly, the article has been retracted, as the editors consider its conclusions invalid. The corresponding author, Yan Yong‐min, agreed with the retraction decision; no confirmation was obtained by the remaining co‐authors.

